# Monitoring the charge-transfer process in a Nd-doped semiconductor based on photoluminescence and SERS technology

**DOI:** 10.1038/s41377-020-00361-0

**Published:** 2020-07-10

**Authors:** Shuo Yang, Jiacheng Yao, Yingnan Quan, Mingyue Hu, Rui Su, Ming Gao, Donglai Han, Jinghai Yang

**Affiliations:** 1 College of Science, Changchun University, Changchun, 130022 China; 2 National Demonstration Centre for Experimental Physics Education, Jilin Normal University, Siping, 136000 China; 3 Key Laboratory of Functional Materials Physics and Chemistry of the Ministry of Education, Jilin Normal University, Changchun, 130012 China; 4 Key Laboratory of Preparation and Application of Environmental Friendly Materials, Jilin Normal University, Ministry of Education, Changchun, 130103 China; 5 School of Materials Science and Engineering, Changchun University of Science and Technology, Changchun, 130022 China

**Keywords:** Optical materials and structures, Optical physics

## Abstract

Surface-enhanced Raman scattering (SERS) and photoluminescence (PL) are important photoexcitation spectroscopy techniques; however, understanding how to analyze and modulate the relationship between SERS and PL is rather important for enhancing SERS, having a great effect on practical applications. In this work, a charge-transfer (CT) mechanism is proposed to investigate the change and relationships between SERS and PL. Analyzing the change in PL and SERS before and after the adsorption of the probe molecules on Nd-doped ZnO indicates that the unique optical characteristics of Nd^3+^ ions increase the SERS signal. On the other hand, the observed SERS can be used to explain the cause of PL background reduction. This study demonstrates that modulating the interaction between the probe molecules and the substrate can not only enhance Raman scattering but also reduce the SERS background. Our work also provides a guideline for the investigation of CT as well as a new method for exploring fluorescence quenching.

## Spectroscopy: rare earths help sensors focus on the necessities

Sensors that use laser light scattering to detect molecular-scale changes to samples can be improved with a new substrate that naturally reduces background interference. Surface-enhanced Raman spectroscopy (SERS) setups typically use a combination of metal nanoparticles and fluorescent probes to capture vibrational signals from biomolecules including DNA. Ming Gao from Jilin Normal University in Siping, China, and colleagues now report a technique to keep probe emissions from interfering with SERS signals. To accomplish this, the team synthesized a neodymium-doped zinc oxide spiky nanostructures substrate. Spectroscopic measurements revealed that neodymium’s electronic properties enabled it to transfer photoexcited electrons to the probes. This charge transfer reduced the probe’s fluorescent intensity and simultaneously enhanced SERS emissions beyond that available on pure zinc oxide surfaces.

## Abstract (web summary)

This study provides a new way to naturally eliminate the SERS fluorescence background by doping with Nd ions. These fundamental discussions here provide a path to enhance Raman scattering and reduce the SERS background and a guideline for the investigation of CT and a new method for exploring fluorescence quenching.

## Graphical abstract

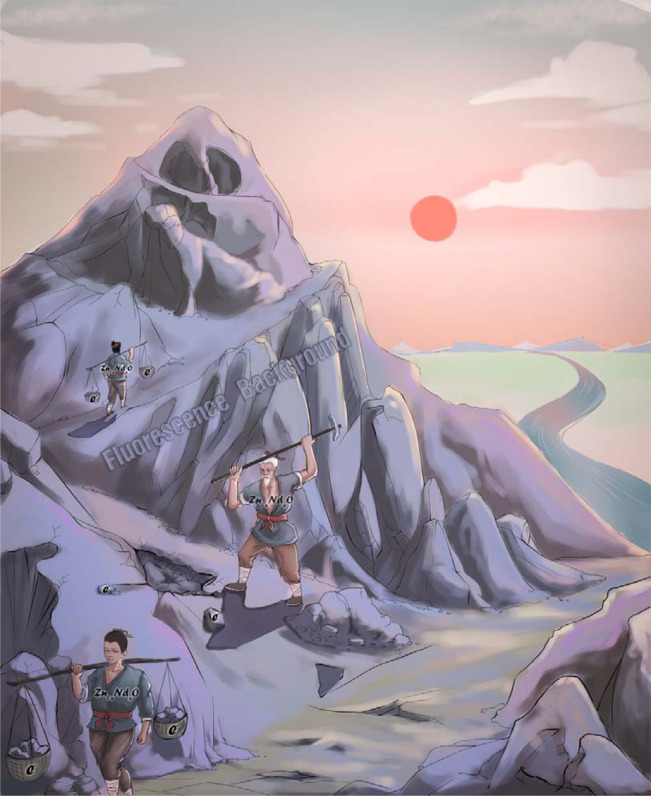

## Introduction

Surface-enhanced Raman scattering (SERS), as a powerful spectral technology, has been widely used in the fields of chemistry, pharmaceuticals, biosensors, food detection, and environmental monitoring owing to its high sensitivity and fast response^1–3^. In general, the enhanced magnitude of SERS is associated with two mechanisms. One is the electromagnetic mechanism, which is related to the localized surface plasmon resonance of the metal nanoparticles (NPs)^4^. The other is the chemical mechanism, which mainly originates from the charge transfer (CT) between adsorbed molecules and SERS-active substrates^5^. On the other hand, the photoluminescence (PL) of the substrates and adsorbed molecules, acting as a broad-continuum background of SERS spectroscopy, has a great effect on SERS spectroscopy and evenly reduces the distinctiveness of the Raman tag^6,7^. Thus, controlling and utilizing PL, contributed by substrates and adsorbed molecules, to enhance the SERS signal is a key problem that urgently needs to be solved. Recently, Ren et al. successfully resolved these limitations by proposing a method for recovering native chemical information from SERS using plasmonic PL and quantitatively investigated the relationship between the PL and the SERS background^8^. However, the development of simpler and more effective methods to remove the negative effects of PL on SERS and to directly analyze the relationship between SERS and PL is of significance for both fundamental research and practical application.

In the PL generation mechanism, photoexcited electrons transition to a high energy level. Because of high-energy-level instability, these electrons usually transition to a low energy level and emit photons^9,10^. This inspires us to use electrons as a link to explore the relationship between PL and SERS in the CT mechanism and investigate how PL affects the SERS signal. SERS technology has been widely used to monitor the charge transport of substrate–molecular junctions^11,12^. Generally, when a semiconductor is used as a SERS substrate, only the CT enhancement mechanism contributes to SERS signals. However, exploring the relationship between SERS and PL of pure semiconductors is not obvious and thus is not convenient to analyze. Previously, we introduced impurity ions to optimize the matrix semiconductor^10,13^. The intra-4*f* emission spectra of Nd^3+^ are characterized by narrow lines with high color purity because the 4*f* electrons of rare-earth ions are shielded from external forces by the outer 5*s* and 5*p* electrons^14^. Thus, incorporating Nd^3+^ ions into a semiconductor might be an effective way to enhance the SERS signal. On the other hand, the characteristic PL peaks of Nd^3+^ ions can be used to analyze the relationship between PL and SERS. In this study, the nanomaterial ZnO was selected as a SERS substrate owing to its good optical stability and relatively high SERS activity among the reported semiconductor nanomaterials^15,16^.

In this work, we successfully synthesized Nd-doped ZnO (Zn_1 − *x*_Nd_*x*_O) as a SERS substrate in which Nd doping was performed using a simple chemical method. Here, we creatively used the CT mechanism to establish the relationship between SERS and PL and examined the change in SERS and PL caused by the CT mechanism in detail. We found that both Nd^3+^ ions and probe molecule fluorescence quenching promote CT, enhance the SERS effect, and reduce the SERS background. This is thus the first example of using PL to enhance SERS and provides a new method for exploring fluorescence quenching.

**Fig. 1:**
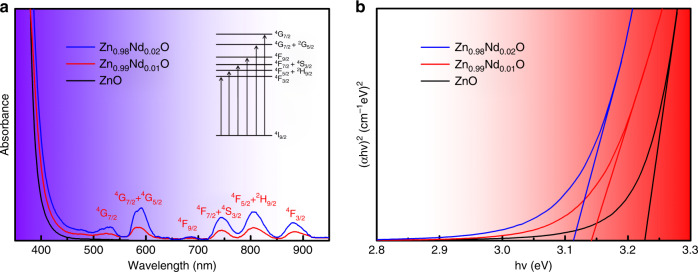
The UV-vis absorption spectra and of Optical energy band gap ZnO and Zn_1−*x*_Nd_*x*_O. **a** The UV–vis absorption spectra of ZnO and Zn_1−*x*_Nd_*x*_O (*x* = 0.00, 0.01, 0.02). **b** Optical energy band gap of ZnO and Zn_1−*x*_Nd_*x*_O (*x* = 0.00, 0.01, 0.02)

Zn_1 − *x*_Nd_*x*_O (*x* = 0.00, 0.005, 0.01, 0.015, 0.0175, 0.02, 0.0225, and 0.03) was prepared via the coprecipitation method (details regarding the synthesis are presented in the Supporting Information, Fig. S1). X-ray diffraction (XRD) patterns (Fig. S2) revealed that the solubility limit of Nd^3+^ ions is ~0.02 in the Zn_1 − *x*_Nd_*x*_O structure and that excessive doping causes the precipitation of Nd_2_O_3_ NPs^17^. The morphologies of ZnO and Zn_0.98_Nd_0.02_O were examined via scanning electron microscopy (SEM) and transmission electron microscopy (TEM). The SEM images show that pure ZnO is a NP, aggregating with a diameter of ~100 nm (Fig. S3a); after Nd doping, Zn_0.98_Nd_0.02_O is a 3D urchin-like nanostructure with a diameter of 2 μm (Fig. S3c). Figure S3b, d (e.g., TEM images) shows that the surface area increases after doping, which improves the adsorption capacity. Among these eight samples with different doping amounts, we selected 0, 1, and 2% representative data for subsequent research. The UV–vis absorption spectra of Zn_1 − *x*_Nd_*x*_O (*x* = 0.00, 0.01, 0.02) are shown in Fig. 1a. It is noteworthy that there are some characteristic peaks of Nd^3+^ ions at 879.4, 805.2, 742.5, 685.1, 578.2, and 521.0 nm corresponding to intra-4*f* shell electron transitions of Nd^3+^ ions^18^, as shown in the inset of Fig. 1a. It is well known that optical absorption properties are associated with the optical band gap (Eg), which can be obtained by Tauc’s formula (*αhν*)^2^ = *hν* − Eg, as shown in Fig. 1b^19^. The band gaps of Zn_1 − *x*_Nd_*x*_O (*x* = 0.00, 0.01, 0.02) were derived to be 3.23, 3.14, and 3.11 eV, respectively. Zn_1 − *x*_Nd_*x*_O has a smaller band gap value, indicating that it has a preferable optical absorption property, making the interband charge transition easier.

**Fig. 2:**
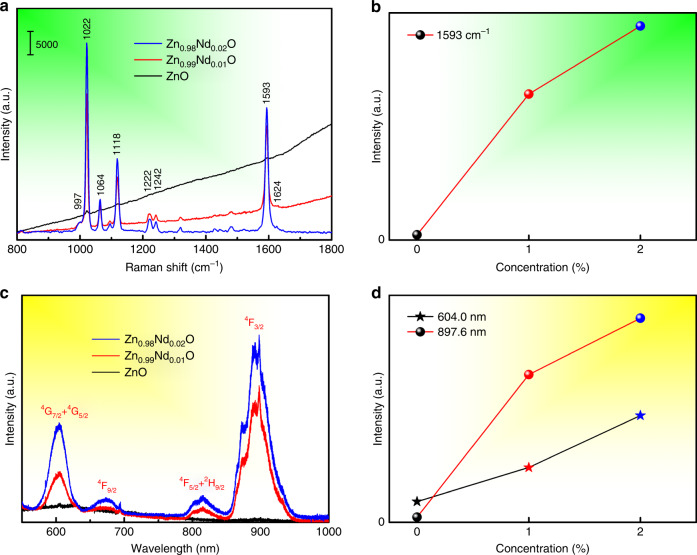
To explore the relationship between SERS and PL, SERS spectra of 4-MPy adsorbed on Zn_1−*x*_Nd_*x*_O and PL spectra of Zn_1−*x*_Nd_*x*_O measured with laser lines of 514.5 nm. **a** SERS spectra of 4-MPy adsorbed on Zn_1−*x*_Nd_*x*_O (*x* = 0.00, 0.01, 0.02) under a 514.5-nm laser. **b** A plot of the SERS intensity of the 1593 cm^−1^ band of 4-MPy versus Nd concentration. **c** PL spectra of Zn_1−*x*_Nd_*x*_O (*x* = 0.00, 0.01, 0.02) under a 514.5-nm laser. **d** A plot of the PL intensity of 604.0 and 897.6 nm versus Nd concentration

To explore the relationship between SERS and PL, we obtained SERS and PL spectra measured with laser lines of 514.5 nm. 4-Mpy (C_5_H_5_NS) was selected as a probe, and the SERS spectra of 4-MPy adsorbed on Zn_1 − *x*_Nd_*x*_O are shown in Fig. 2a (several representative concentrations are shown in Fig. 2a, and other concentrations are given in the Supporting Information, Fig. S4). The SERS spectral signal is enhanced after Nd doping, while the signal contributed by the fluorescence background is weakened. Figure 2b shows the SERS intensity of the 1593 cm^−1^ band of 4-MPy plotted as a function of the Nd concentration. The SERS intensity increases with the Nd concentration and reaches a maximum for Zn_0.98_Nd_0.02_O. Figure 2c displays the PL spectra of Zn_1 − *x*_Nd_*x*_O. The Nd-doped ZnO exhibits dramatically sharp luminescence peaks, which is in sharp contrast with pure ZnO. Moreover, luminescent peaks are located at 897.6, 815.1, 674.2, and 604.0 nm (^4^F_3/2_, ^4^F_5/2_ + ^2^H_9/2_, ^4^F_9/2_, and ^4^G_7/2_ + ^2^G_5/2_), corresponding to the UV–vis absorption spectra^20^. Figure 2d shows the Nd-ion doping concentration-dependent PL intensity of Zn_1 − *x*_Nd_*x*_O, which initially increases with the enhancement of the Nd concentration. Notably, the change in the PL intensity (Fig. 2d) is consistent with the change in the SERS intensity (Fig. 2b).

**Fig. 3:**
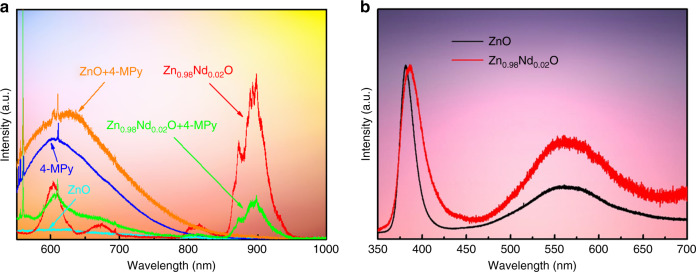
PL spectra of Zn_1−*x*_Nd_*x*_O with and without 4-MPy molecules upon 514.5 nm and 325 nm laser excitation. **a** PL spectra of ZnO, Zn_0.98_Nd_0.02_O, 4-MPy, 4-MPy+ZnO, and 4-MPy+Zn_0.98_Nd_0.02_O upon 514.5-nm laser excitation; **b** PL spectra of ZnO, Zn_0.98_Nd_0.02_O, and ZnO upon 325-nm laser excitation

To further investigate the relationship between the PL and SERS of Zn_1 − *x*_Nd_*x*_O, we examined the change in the PL spectrum of Zn_1 − *x*_Nd_*x*_O (*x* = 0.00, 0.01, 0.02) with and without 4-MPy molecules. Figure 3a shows that the PL signal of 4-MPy + ZnO is a simple superposition of the fluorescence of ZnO and 4-MPy, but the PL signal of 4-MPy + Zn_0.98_Nd_0.02_O is reduced in comparison with that of 4-MPy molecules. The intensities of the luminescence peaks at 897.6 and 815.1 nm (^4^F_3/2_ and ^4^F_5/2_ + ^2^H_9/2_) significantly decrease, while that of the luminescence peak at 604.0 nm (^4^G_7/2_ + ^2^G_5/2_) shows a relatively small decrease. In addition, the SERS signal of 4-MPy + Zn_0.98_Nd_0.02_O is significantly increased (Fig. 2a), indicating that there is obvious CT between Zn_0.98_Nd_0.02_O substrates and 4-MPy molecules.

To probe the CT mechanism between Zn_1 − *x*_Nd_*x*_O (*x* = 0.00, 0.01, 0.02) and 4-MPy in the SERS spectrum, first, UPS and UV–vis spectra were used to determine the position of each energy level. The highest occupied molecular orbital (HOMO) and lowest unoccupied molecular orbital (LUMO) levels of 4-MPy are −9.77 and −6.34 eV, respectively (calculated from Figs. S5 and S6). The maximum valence band (VB) and minimum conduction band (CB) of Zn_0.98_Nd_0.02_O are −9.11 and −6.00 eV (calculation process in SI, Fig. S7), respectively. Figure 3b shows the PL spectra of pure ZnO and Zn_0.98_Nd_0.02_O upon 325-nm laser excitation. The two samples consist of two emission bands: a near band edge at ~383 nm and a wide deep level emission (DLE) from 480 to 660 nm^21^. The DLE is attributed to intrinsic defects, such as oxygen vacancies or various surface states^22^. On the other hand, Nd doping increases the number of surface defects of ZnO. These oxygen vacancies and surface defects induce new surface state energy levels (Ess) of 1.88–2.58 eV^13^, located above the top of the VB (Fig. 3b). In addition, theoretical calculations indicate that the electronic ground state (^4^I_9/2_) of the Nd^3+^ ions is located ~1 eV below the top of the VB^23^. Thus, the excited-state energy levels of the Nd^3+^ ions are located at −8.73, −8.61, −8.27, and −8.06 eV, respectively.

**Fig. 4:**
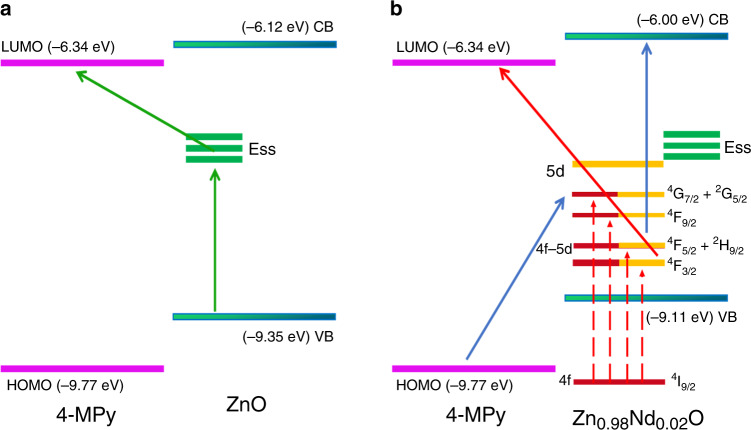
Schematic representations for charge-transfer mechanisms between **a** 4-MPy and ZnO and **b** 4-MPy and Zn_0.98_Nd_0.02_O

Based on the above results, we analyzed the enhancement mechanism of Zn_1 − *x*_Nd_*x*_O (*x* = 0.00, 0.01, 0.02, taking Zn_0.98_Nd_0.02_O as an example). As shown in Fig. 4a, upon excitation at 514.5 nm (2.41 eV), the VB electrons of ZnO can be excited to Ess, transition to the LUMO level of the adsorbed 4-MPy molecules, and finally return to the VB of ZnO to release a Raman photon^24^. Considering that pure ZnO contains few oxygen defects, ZnO only undergoes CT in this process. Therefore, its SERS intensity is very low, and the PL signal with the probe molecules is superimposed.

As shown in Fig. 4b, for Zn_0.98_Nd_0.02_O, the 514.5-nm laser is able to excite the electrons of the 4*f* shell of the Nd^3+^ ions from the ground state (^4^I_9/2_) to the excited states (^4^F_3/2_, ^4^F_5/2_ + ^2^H_9/2_, ^4^F_9/2_, and ^4^G_7/2_ + ^2^G_5/2_). Then, the excited-state electrons transfer to the LUMO level and finally return to the ground state of the Nd^3+^ ions, releasing Raman photons. CT from the excited-state electrons of the Nd^3+^ ions to the 4-MPy molecule reduces the number of electrons returning to the ground state, resulting in fluorescence quenching of Nd^3+^ ions. The energy required for transition from the excited-state ^4^F_3/2_ to the LUMO is 2.39 eV, and the laser energy of 514.5 nm is exactly 2.41 eV. The two almost identical energy levels induce the occurrence of CT resonance. When ^4^F_5/2_ + ^2^H_9/2_ transitions to a higher unoccupied molecular orbital, CT resonance can also occur. Thus, the luminescence peaks (^4^F_3/2_ and ^4^F_5/2_ + ^2^H_9/2_) significantly decrease. However, the energy required to transfer electrons at ^4^G_7/2_ + ^2^G_5/2_ to the 4-MPy molecule is much lower in comparison with that for ^4^F_3/2_. The CT resonance cannot occur, resulting in a lower probability of CT. As a consequence, the intensity of the ^4^G_7/2_ + ^2^G_5/2_ luminescence peak has only a relatively small decrease. To better prove this electron transfer between Zn_0.98_Nd_0.02_O and 4-MPy, we measured the fluorescence lifetime of Zn_0.98_Nd_0.02_O before and after absorbing the 4-MPy molecule and collected their emission decays at ^4^G_7/2_ + ^2^G_5/2_ (Fig. S8a) and ^4^F_3/2_ (Fig. S8b). It was found that when the 4-MPy molecule is adsorbed, the lifetime of the ^4^F_3/2_ energy level (897.6 nm) decreases rapidly, whereas the lifetime of the ^4^G_5/2_ + ^2^G_7/2_ energy level (604.0 nm) remains almost unchanged. Therefore, the fluorescence quenching of Nd^3+^ ions significantly increases the SERS intensity of Zn_1 − *x*_Nd_*x*_O, realizing that the utilization of unique optical characteristics of Nd^3+^ ions promotes the SERS signal. As shown in the theoretical calculation (Fig. S9), the empty Nd 4*f* impurity levels are close to the Nd 5*d* levels, leading to mixing of the 4*f* and small amounts of the 5*d* orbitals, referred to as the 4*f*–5*d* orbital. Under laser irradiation, the 4*f* ground-state electrons of Nd^3+^ ions can be excited into the higher empty 4*f*–5*d* levels, and then these excited 4*f*–5*d* electrons jump into the 5d levels under continuous irradiation^25^. At the same time, the electrons in the 5*d* orbital are reductive and easily lose electrons under irradiation by light^26^. Overall, the CT channel, originating from charge-transfer resonance, overcomes the effect of the 4*f* levels shielded by external 5*s* and 5*p* electrons.

Furthermore, upon laser excitation, the HOMO-level electrons of the 4-MPy molecules transfer to the excited states and then to the CB of Zn_1 − *x*_Nd_*x*_O. They finally return to the HOMO level of the 4-MPy molecules, releasing Raman photons. This process inhibits the recombination of electrons and holes in the HOMO level, resulting in fluorescence quenching of 4-MPy molecules. The above CT processes work together toward the enhancement of SERS signals and significantly reduce the fluorescence background.

**Fig. 5:**
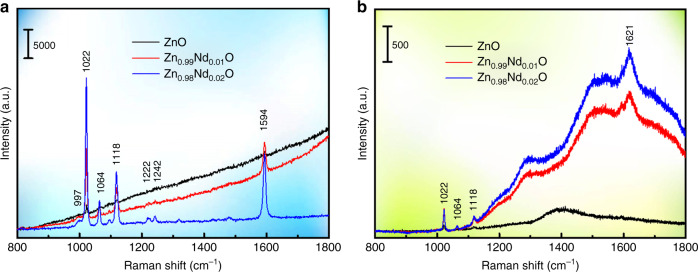
SERS spectra of 4-MPy adsorbed on Zn_1 − *x*_Nd_*x*_O (*x* = 0.00, 0.01, 0.02) under different laser excitation: **a** 633 nm and **b** 785 nm

To further verify these CT processes, the SERS spectra of Zn_1 − *x*_Nd_*x*_O (*x* = 0.00, 0.01, 0.02) were analyzed under 633 and 785 nm laser irradiation (Fig. 4). The SERS spectra at 633 nm irradiation were essentially the same as the spectrum at 532 nm irradiation (Fig. 5a). However, the SERS spectra at 785 nm irradiation were significantly different (Fig. 5b): the SERS background increased significantly, while the SERS intensity was only slightly enhanced. The 633-nm laser (1.96 eV) can maximally excite electrons from ^4^I_9/2_ to ^4^F_9/2_ (1.84 eV) and then transfer to the LUMO level (1.93 eV). However, the 785-nm laser (1.58 eV) preferentially excites electrons from ^4^I_9/2_ to ^4^F_5/2_ + ^2^H_9/2_ (1.52 eV), but the laser energy is not sufficient to again excite the electrons to the LUMO level (2.27 eV). In the case of an increase in the PL of Nd-doped ZnO, the inability of the substrate to reduce the PL signal via CT is responsible for the SERS background enhancement. Although the SERS spectra obtained under different excitation lines are different, they all support our proposed mechanism.

In summary, we analyzed the change between the PL and SERS relative intensities of Nd-doped ZnO before and after the adsorption of probe molecules to explore the CT mechanisms in Nd-doped ZnO systems. The results indicated that the unique CT between Nd^3+^ ions and probe molecules improves the SERS performance and naturally eliminates the SERS fluorescent background. Moreover, the mechanism is further confirmed by examining the SERS spectra under various excitation wavelengths. This work paves the way for developing novel molecular-sensing techniques.

## Methods

Zn_1 − *x*_Nd_*x*_O was synthesized using the coprecipitation method. In brief, Zn(NO_3_)_2_·6H_2_O and Nd(NO_3_)_3_·6H_2_O were dissolved in deionized water with a molar ratio where Nd/(Nd + Zn) was *x*:1 (*x* = 0.00, 0.005, 0.01, 0.015, 0.0175, 0.02, 0.0225, 0.03). After stirring for 20 min, NH_4_HCO_3_ aqueous solution was added. After stirring for 4 h, the white precipitates were collected by centrifugation, washed with deionized water and ethanol several times, and then dried under vacuum at 80 °C for 12 h. Finally, the sample was further annealed in air for 1 h at 600 °C to obtain the final Zn_1 − *x*_Nd_*x*_O (*x* = 0.00, 0.005, 0.01, 0.015, 0.0175, 0.02, 0.0225, and 0.03) products.

We evaluated the structural quality of the samples with X-ray diffraction (XRD, Rigaku D/Max 3C). X-ray photoelectron spectroscopy (XPS, VG ESCALAB 250X) was used to analyze the element content of the samples. The morphology was characterized by field emission SEM (JEOL JSM-6700F) and TEM (JEM-2100HR). UV–vis absorption spectra were measured by a Shimadzu 3600 spectrometer. Under a 514.5 nm (2.41 eV) Ar^+^ ion laser, the Renishaw inVia Raman system detected all SERS and PL signals with a laser power of 40 mW, attenuation of 100%, 10-s exposure time, and 1 scan.

## Supplementary information

Supplementary information
