# Supplementary material for: Monitoring the charge-transfer process in a Nd-doped semiconductor based on photoluminescence and SERS technology
Source: Light Sci Appl. 2020 Jul 10;9:117. doi: 10.1038/s41377-020-00361-0 (PMC7351777; doi:10.1038/s41377-020-00361-0)
Supplement: Supplementary file 1 — Supplementary information [file 41377_2020_361_MOESM1_ESM.doc]

Supplementary information for

**Monitoring the Charge Transfer Process in a Nd-doped Semiconductor Based on Photoluminescence and SERS Technology**

**Shuo Yang^1^, Jiacheng Yao^2, 3, 4^, Yingnan Quan^2, 3, 4^, Mingyue Hu^2, 3, 4^, Rui Su^2, 3, 4^, Ming Gao^2, 3, 4^*, Donglai Han^5^*, and Jinghai Yang^2, 3, 4^***

^1^College of Science, Changchun University, Changchun, 130022, China.

^2^National Demonstration Centre for Experimental Physics Education, Jilin Normal University, Siping 136000, China.

^3^Key Laboratory of Functional Materials Physics and Chemistry of the Ministry of Education, Jilin Normal University, Changchun 130012, China.

^4^Key Laboratory of Preparation and Application of Environmental Friendly Materials, Jilin Normal University, Ministry of Education, Changchun, 130103, China.

^5^School of Materials Science and Engineering, Changchun University of Science and Technology, Changchun, 130022, China.

*Corresponding author: Ming Gao, Donglai Han, Jinghai Yang

*E-mail address:* [gaomingphy@126.com (M. Gao);](mailto:gaomingphy@126.com;) [DLHan_1015@163.com](mailto:DLHan_1015@163.com) (D. L. Han); jhyang1@jlnu.edu.cn (J. H. Yang)


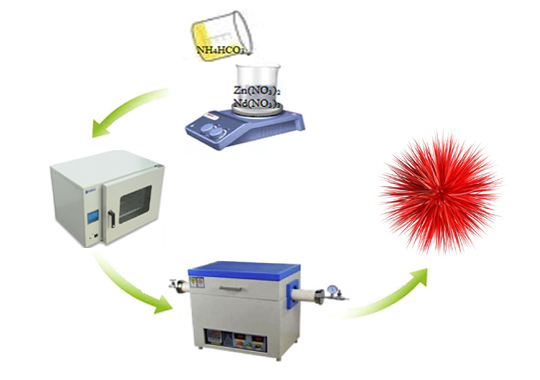


**Figure S1.** Schematic illustration of the details synthetic process.


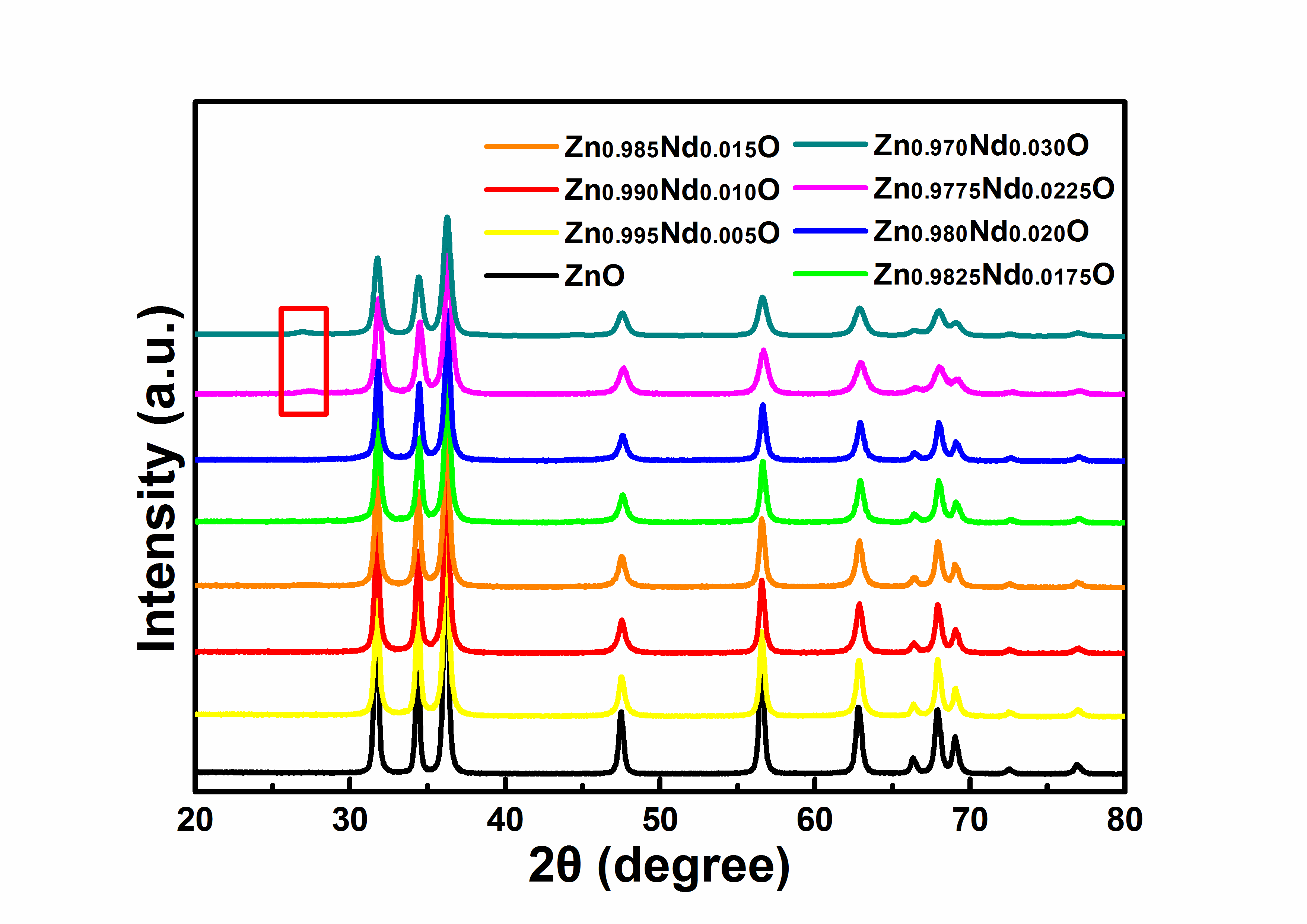


**Figure S2.** (a) The XRD pattern of the Zn_1-x_Nd_x_O (x=0.00, 0.005, 0.01, 0.015, 0.0175, 0.02, 0.0225, 0.03).


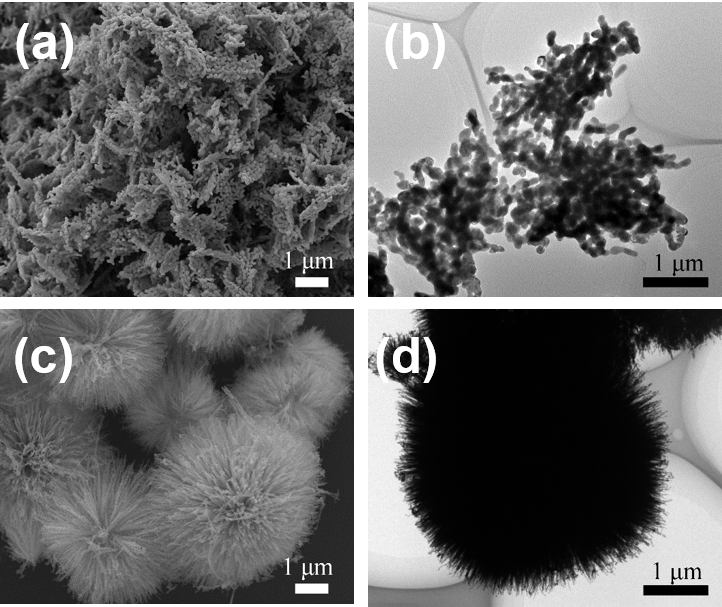


**Figure S3.** (a) SEM image of ZnO, (b) TEM image of Zn_0.98_Nd_0.02_O, (c) SEM image of ZnO, (d) TEM image Zn_0.98_Nd_0.02_O.


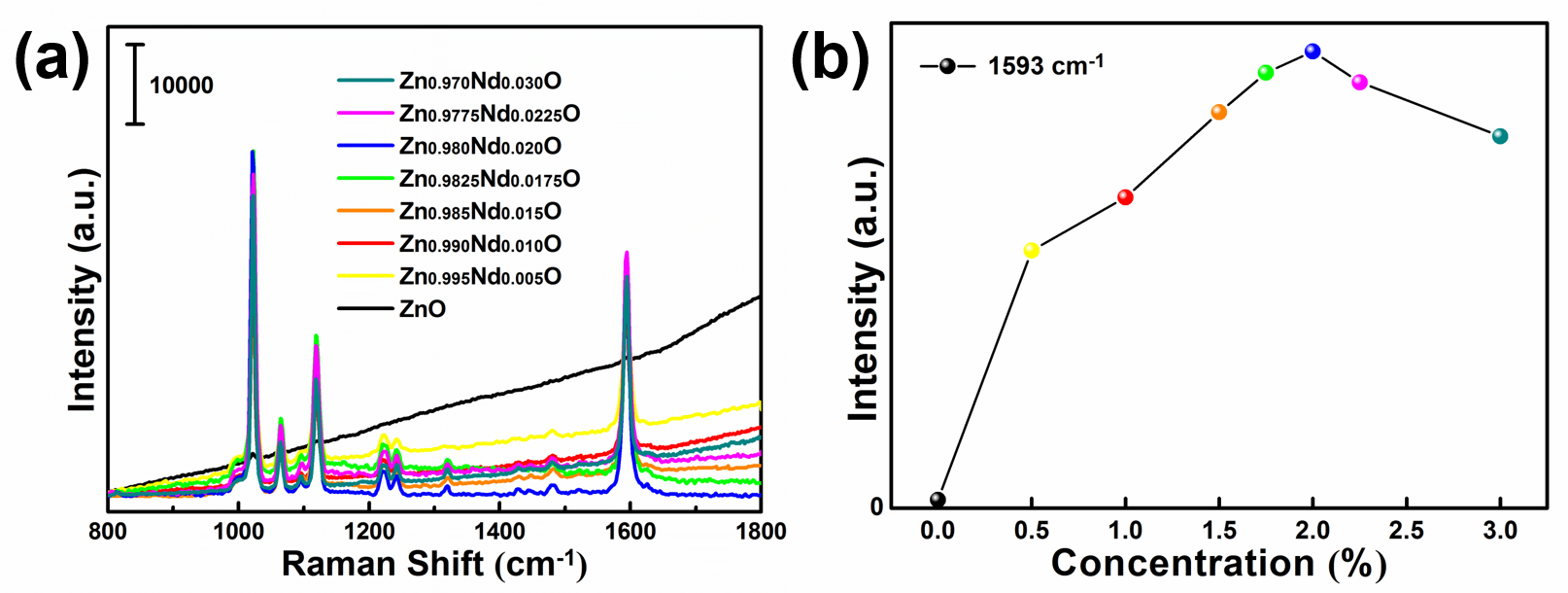


**Figure S4.** (a) SERS spectra of 4-MPy adsorbed on Zn_1-x_Nd_x_O (x=0.00, 0.005, 0.01, 0.015, 0.0175, 0.02, 0.0225, and 0.03) under 514.5 nm laser. (b) A plot of the SERS intensity of the 1593 cm^-1^ band of 4-MPy versus Nd concentration.

**
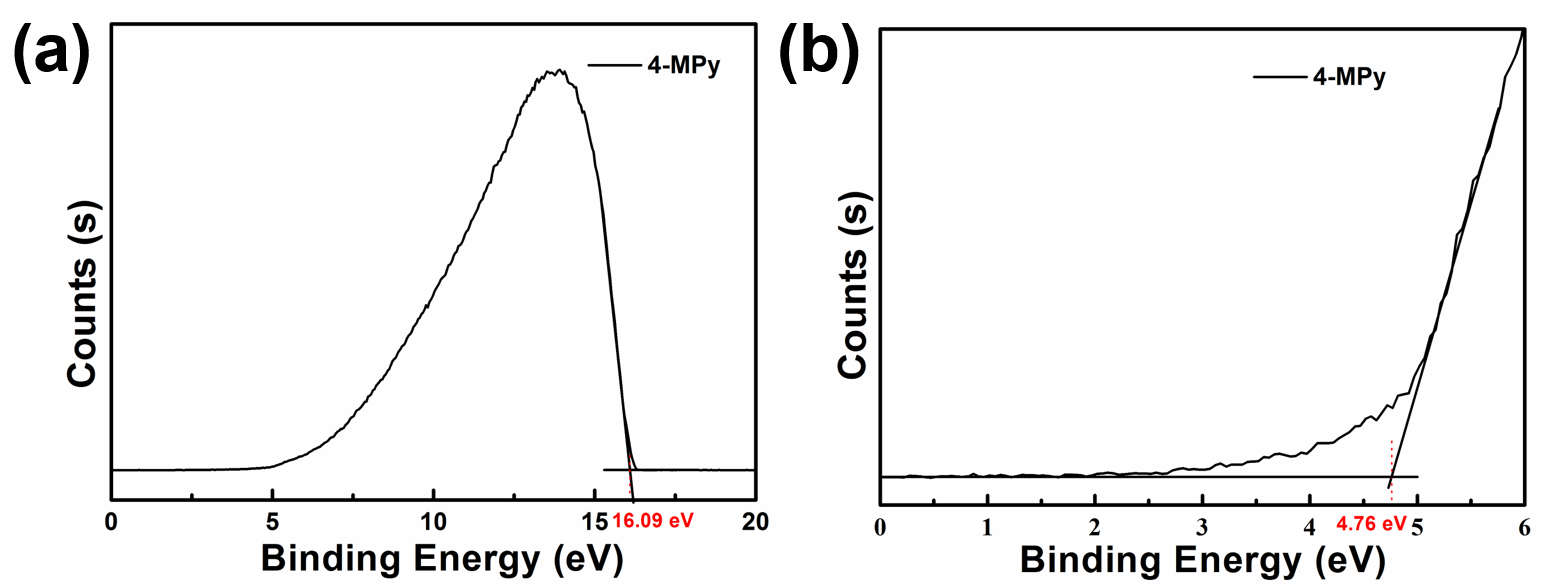
**

**Figure** **S5.** The UPS of 4-MPy molecule: (a) 0-22 eV; (b) 0-6 eV.


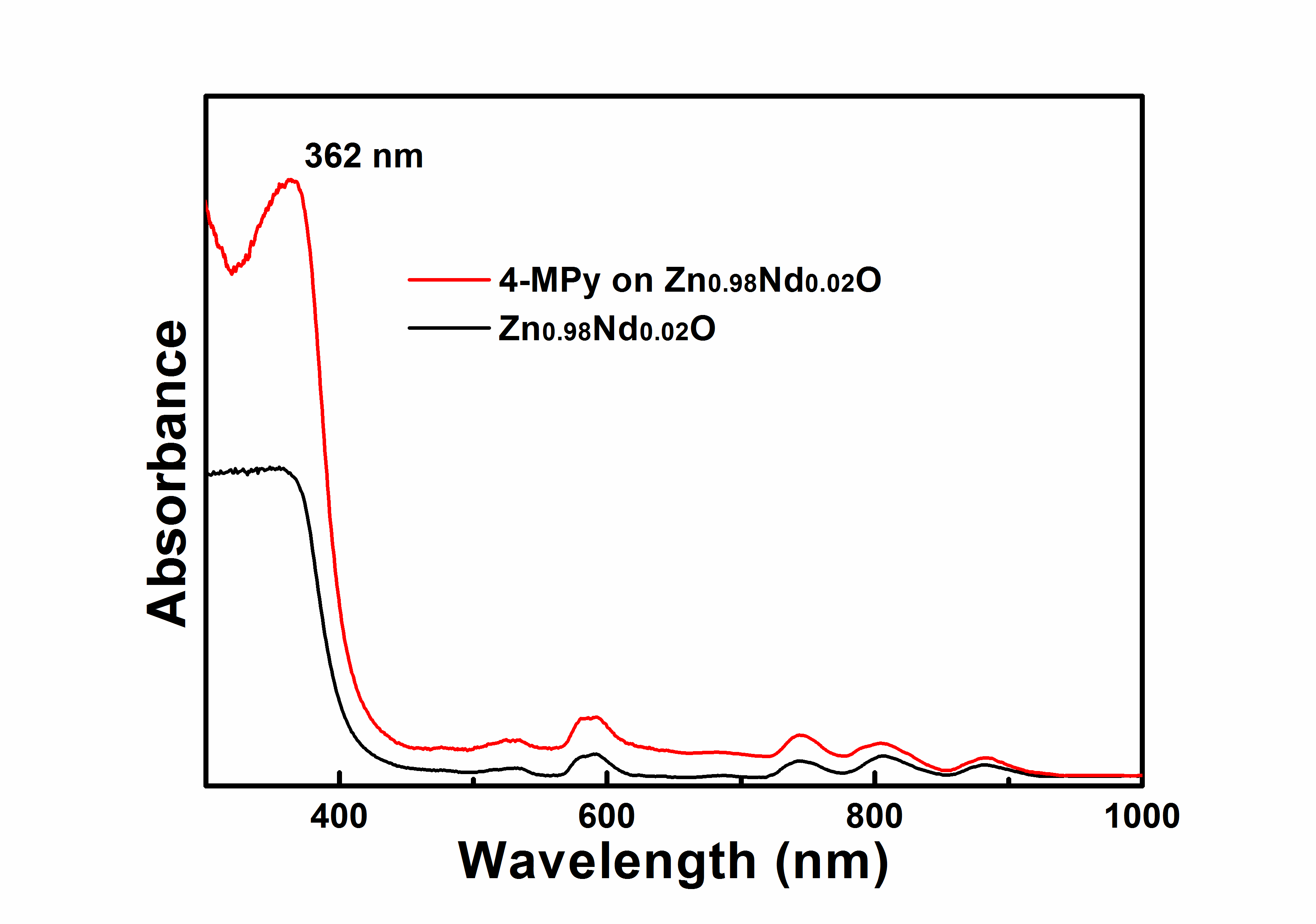


**Figure S6.** The UV-vis spectrum of 4-MPy molecule.

The work function formula: W=*hν*-ΔE_a_, among them *hν*=21.22 eV, and the HOMO formula: HOMO= -(W+ΔE_b_). According to Figure S5, the work function of 4-Mpy is 5.13 eV and the HOMO of 4-Mpy is situated at -9.77 eV. As shown in Figure S6, the UV-Vis absorption maximum is located at 362 nm, and thus the band gap of 4-Mpy between HOMO and LUMO is 3.43 eV. The LUMO of 4-Mpy is situated at -6.34 eV.


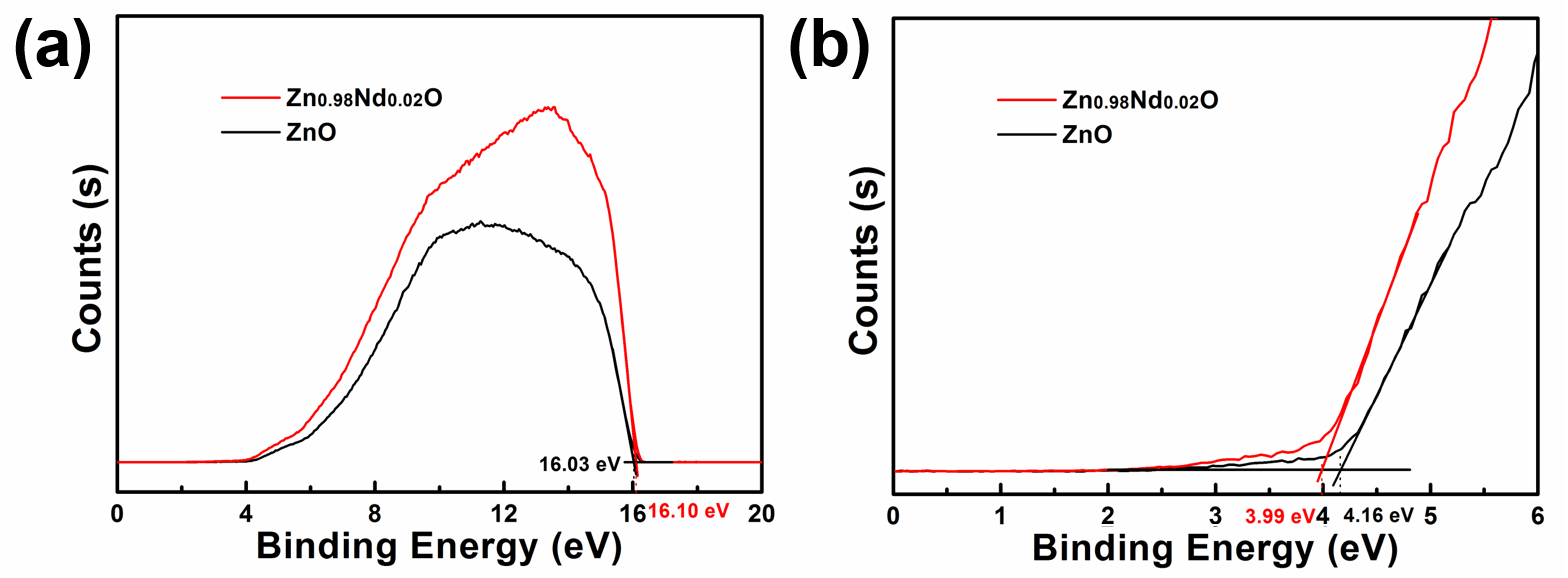


**Figure S7.** The UPS of ZnO and Zn_0.98_Nd_0.02_O substrates: (a) 0-22 eV; (b) 0-6 eV

According to Figure S7, the work function of Zn_1-x_Nd_x_O is 5.12 eV and the VB of Zn_0.98_Nd_0.02_O is situated at -9.11eV. As band gap is between VB and CB is 3.11 eV, and thus the CB of ZNO is situated at -6.00 eV. In a similar way, the work function, VB and CB of ZnO is 5.19, -9.35 and -6.12 eV, respectively.


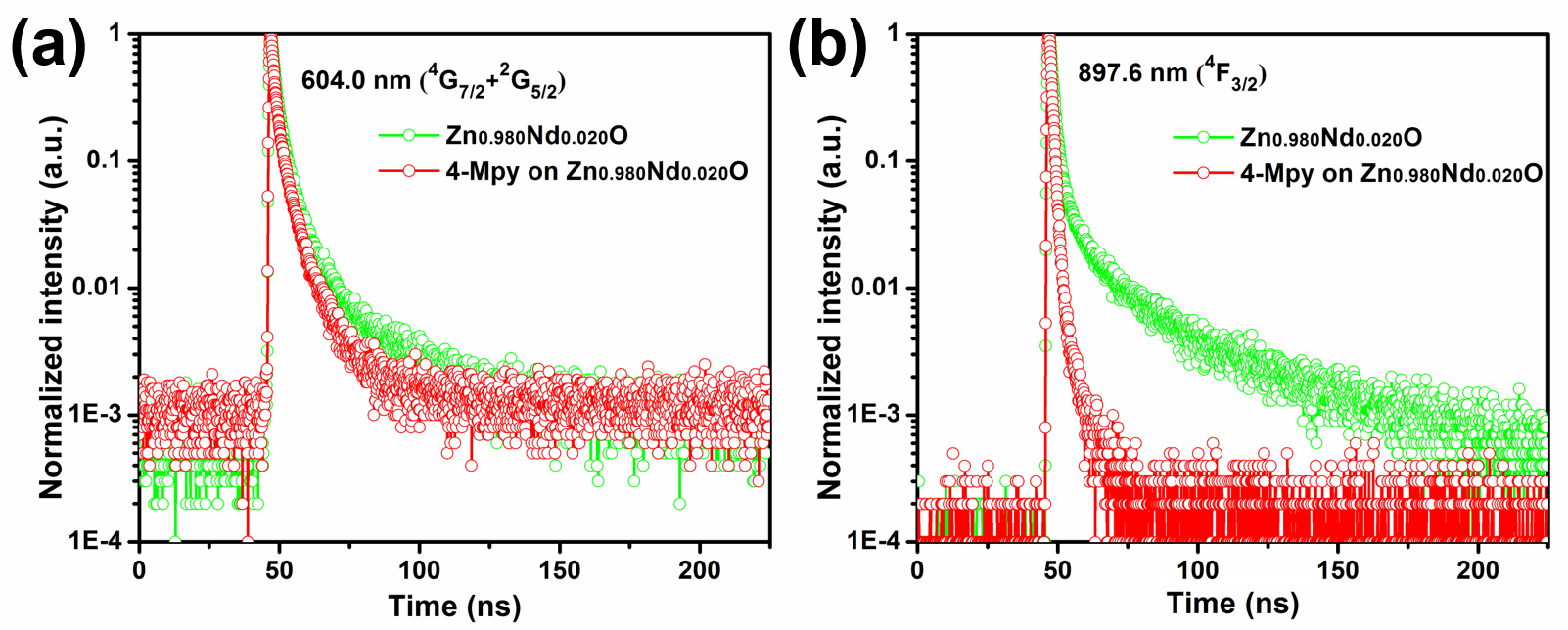


**Figure S8.** The fluorescence lifetime of PL peaks: (a) 604.0 nm; (b) 897.6 nm.


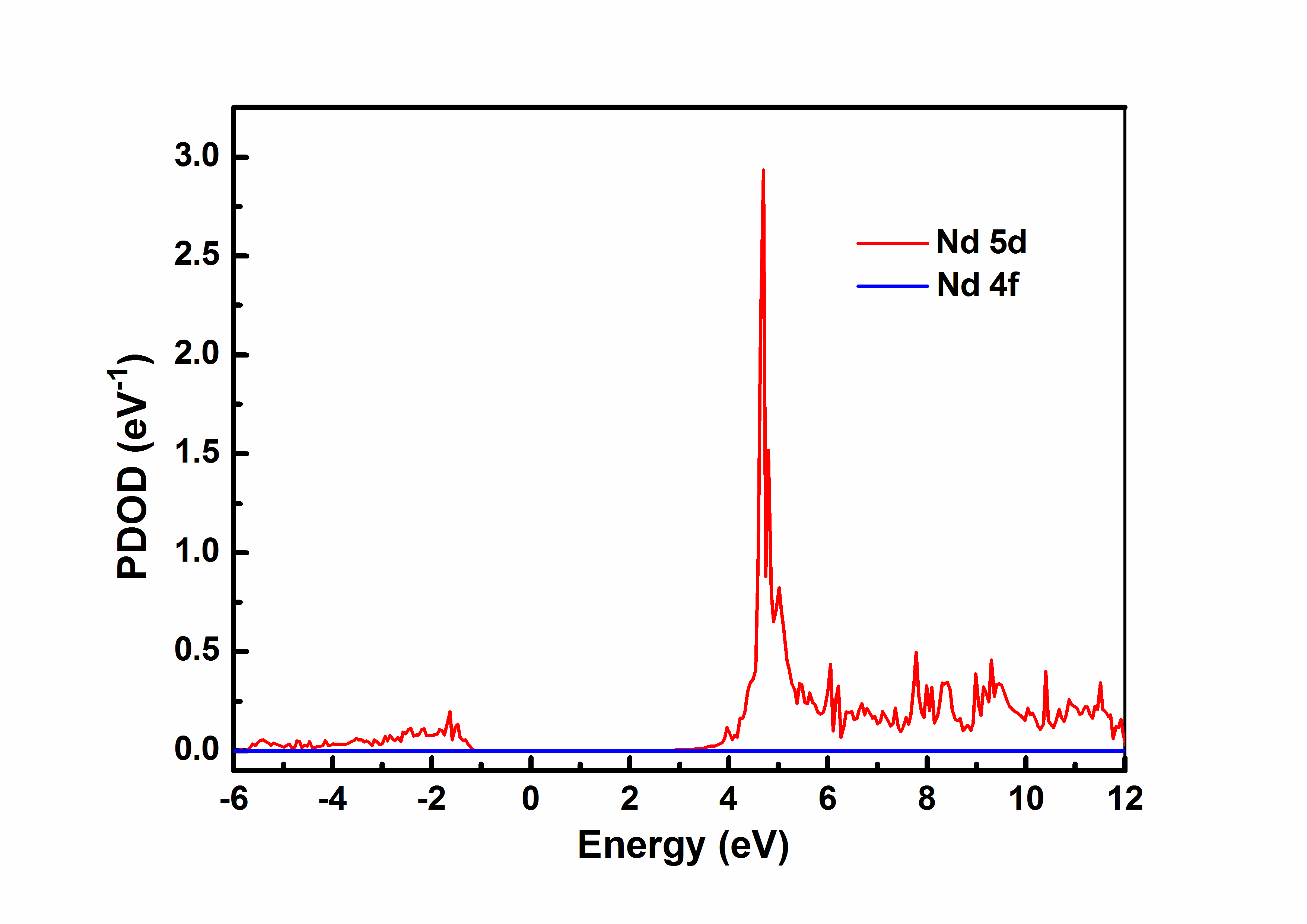


**Figure S9.** Calculated PDOS of Zn_0.98_Nd_0.02_O substrates. The Fermi level is set to 0 eV and indicated by a black dashed line

All the spin theoretical simulations in our work were carried out on the Vienna ab initio Simulation Package (VASP) with the version 5.4.1^1^. The Generalized gradient approximation (GGA) with the Perdew-Burke-Emzerhof (PBE)^2^ functional form was employed to evaluate the electron-electron exchange and correlation interactions while the projector augmented-wave (PAW) methods^3^ were implemented to represent the core-electron (valence electron) interactions. The GGA+U^4^ calculation were performed with the on-site Coulomb repulsion U term on the Zn 3d and Nd 4f states and the U_eff_ (U_eff_=U-J) values are 7.50 eV and 6.622 eV (U=7.609 eV, and J=0.987eV)^5^, respectively. Plane-Wave basis function was set with a kinetic cut-off energy of 400 eV. The ground-state atomic geometries were optimized by relaxing the force to below 0.02 eV/Å and the convergence criteria for energy was set with the value of 1.0
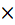
 10^-5^ eV/cell. A Monkhorst-Pack meshes^6^ with the size of 3
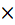
 3
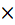
 3 was employed to sample the bulk Brillouin zone for the electronic properties. Tetrahedron method with Blöchl corrections^7^ was employed for the electronic structures and the total energy of our models while the Gaussian smearing was set? for stress/force relaxations. A 3
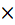
 3
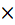
 3 supercell of ZnO was employed for our simulations.

1. G. Kresse, D. Joubert, Phys. Rev. B 59 (1999) 1758. 8
2. J. Perdew, K. Burke, M. Ernzerhof, Phys. Rev. Lett. 77 (1996) 3865. 9
3. P. E. Blohl, Phys. Rev. B: Condens. Matter Mater. Phys., 1994, 50, 17953–17979.
4. A. Liechtenstein, V. Anisimov, J. Zaanen, Phys. Rev. B 52 (1995) R5467.
5. X.J. Zhang, W.B. Mi, X.C. Wang, H.L. Bai, J. Alloys. Compd. 617 (2014) 25. 828-833
6. H. J. Monkhorst and J. D. Pack, Phys. Rev. B: Solid State, 1976, 13, 5188–5192.
